# Supplementary material for: Associations of frailty with partial and absolute sedentary behaviours among older adults: A STROBE-compliant analysis of modifiability by gender and age
Source: PLoS One. 2023 Oct 26;18(10):e0293482. doi: 10.1371/journal.pone.0293482 (PMC10602243; doi:10.1371/journal.pone.0293482)
Supplement: S1 Appendix — (ZIP) [file pone.0293482.s001.zip › Appendix A.doc]

Appendix A. Measures of Sedentary behaviour

The following items describe your sitting habits. In the last 7 days, how much **time (in minutes)** did you spend on each habit on a typical day? Please insert **0** or **NIL** if you didn’t perform the habit.

| No. | Habit | Time (minutes) |
| --- | --- | --- |
| 1 | Sitting for hobbies or playing indoor games in-person (e.g., playing Ludo, Oware, cards) |  |
| 2 | Sitting while using the phone, computer, or internet |  |
| 3 | Sitting while doing household tasks (slicing onions) |  |
| 4 | Sitting while caring for others (e.g., babies) |  |
| 5 | Sitting while eating and drinking |  |
| 6 | Driving a car or riding a motorcycle |  |
| 7 | Sitting in a car as a passenger |  |
| 8 | Sitting while viewing the television |  |
| 9 | Listening to radio or any form of audio (e.g., music) when sitting or lying down |  |
| 10 | Sitting while reading (a book, newspaper, magazine) |  |
| 11 | Sitting idle or reclining |  |
| 12 | Lying down to rest (but keeping awake) |  |

Note: Items 1-6 measured partial sedentary behaviour whereas items 7-12 measured absolute sedentary behaviour
