# Supplementary material for: Associations of frailty with partial and absolute sedentary behaviours among older adults: A STROBE-compliant analysis of modifiability by gender and age
Source: PLoS One. 2023 Oct 26;18(10):e0293482. doi: 10.1371/journal.pone.0293482 (PMC10602243; doi:10.1371/journal.pone.0293482)
Supplement: S1 Appendix — (ZIP) [file pone.0293482.s001.zip › Appendix B.doc]

Appendix B. Measures of Frailty

The following questions describe or relate to your level of frailty. Please how would you respond to these questions, taking into account your current physical functional ability?

| No. | Question | Response | |
| --- | --- | --- | --- |
|  |  | No (0) | Yes (1) |
| 1 | Do you feel physically healthy? |  |  |
| 2 | Have you lost a lot of weight recently without wishing to do so? |  |  |
| 3 | Do you experience problems in your daily life due to difficulty in walking? |  |  |
| 4 | Do you experience problems in your daily life due to difficulty maintaining your balance? |  |  |
| 5 | Do you experience problems in your daily life due to poor hearing? |  |  |
| 6 | Do you experience problems in your daily life due to poor vision? |  |  |
| 7 | Do you experience problems in your daily life due to a lack of strength in your hands? |  |  |
| 8 | Do you experience problems in your daily life due to physical tiredness? |  |  |
| 9 | Do you have problems with your memory? |  |  |
| 10 | Have you felt down during the last month? |  |  |
| 11 | Have you felt nervous or anxious during the last month? |  |  |
| 12 | Are you able to cope with problems well? |  |  |
| 13 | Do you live alone? |  |  |
| 14 | Do you sometimes miss having people around you? |  |  |
| 15 | Do you receive enough support from other people? |  |  |

Note: The cut-off point for frailty is 5; frailty increases from 0 to 15.
