# Supplementary material for: Associations of frailty with partial and absolute sedentary behaviours among older adults: A STROBE-compliant analysis of modifiability by gender and age
Source: PLoS One. 2023 Oct 26;18(10):e0293482. doi: 10.1371/journal.pone.0293482 (PMC10602243; doi:10.1371/journal.pone.0293482)
Supplement: S1 Appendix — (ZIP) [file pone.0293482.s001.zip › Appendix C.doc]

Appendix C. Measures of Neighbourhood Walkability

The following statements describe the extent to which your neighbourhood supports walking and other physical activities. On a scale of 1 to 5, where 1 = strongly disagree; 2 = disagree; 3 = somewhat agree; 4 = agree, and 5 = strongly agree, to what extent do you agree or disagree to the following statements?

| # | Statement | 1 | 2 | 3 | 4 | 5 |
| --- | --- | --- | --- | --- | --- | --- |
| 1 | Many places are easy to go within walking distance |  |  |  |  |  |
| 2 | It is easy to walk to a public transport stop |  |  |  |  |  |
| 3 | There are footpaths on most of the streets |  |  |  |  |  |
| 4 | There are crosswalks and pedestrian signals |  |  |  |  |  |
| 5 | The streets in my neighbourhood are not hilly |  |  |  |  |  |
| 6 | Walkers in my neighbourhood can easily be seen |  |  |  |  |  |
| 7 | There is lots of greenery around my neighbourhood |  |  |  |  |  |
| 8 | There are many interesting things to look at |  |  |  |  |  |
| 9 | There is not much traffic along nearby streets |  |  |  |  |  |
| 10 | My neighbourhood has parks and walking trails |  |  |  |  |  |
| 11 | Crime rate in my neighbourhood is not a problem |  |  |  |  |  |
