# Supplementary material for: Associations of frailty with partial and absolute sedentary behaviours among older adults: A STROBE-compliant analysis of modifiability by gender and age
Source: PLoS One. 2023 Oct 26;18(10):e0293482. doi: 10.1371/journal.pone.0293482 (PMC10602243; doi:10.1371/journal.pone.0293482)
Supplement: S1 Appendix — (ZIP) [file pone.0293482.s001.zip › Appendix D.doc]

Appendix D. Measures of Social Network Size

1. In the last 7 days, how many of your social networks (i.e., friends, blood relations, neighbours, workmates, or acquittances) …

| Task | Number |
| --- | --- |
| (i) Visited you by walking, jogging, or bicycling for at least 10 minutes, |  |
| (ii) Accompanied you to exercise (i.e., walk, jog, ride a bicycle) or perform social activities including shopping and recreation for at least 10 minutes. |  |
