# Supplementary material for: Associations of frailty with partial and absolute sedentary behaviours among older adults: A STROBE-compliant analysis of modifiability by gender and age
Source: PLoS One. 2023 Oct 26;18(10):e0293482. doi: 10.1371/journal.pone.0293482 (PMC10602243; doi:10.1371/journal.pone.0293482)
Supplement: S1 Appendix — (ZIP) [file pone.0293482.s001.zip › Appendix E.doc]

Appendix E. Measures against common methods bias

| # | Steps |
| --- | --- |
| Steps at the stage of study design | |
| 1 | Questionnaire was structured to make each section distinct |
| 2 | Instructions for completing each section of the questionnaire were provided in the questionnaire |
| 3 | Research assistants and researchers provided clarifications to the participants when required |
| Statistical technique | |
| 4 | Exploratory factor analysis (with varimax rotation) was used to explore the factor structure of the scales following the one-factor method |
| 5 | Common methods bias is absent if each scale produced more than a factor. |
| 6 | Each scale produced a factor solution with at least 2 factors as follows: frailty (4 factors, total variance = 60.3%, factor loading ≥0.5) and walkability (3 factors, total variance = 71.8%, factor loading ≥0.5) |

**Note:** The above steps were adopted from Asiamah et al. (2022) as well as Jakobsen and Jensen (2015)
