# Supplementary material for: Associations of frailty with partial and absolute sedentary behaviours among older adults: A STROBE-compliant analysis of modifiability by gender and age
Source: PLoS One. 2023 Oct 26;18(10):e0293482. doi: 10.1371/journal.pone.0293482 (PMC10602243; doi:10.1371/journal.pone.0293482)
Supplement: S1 Appendix — (ZIP) [file pone.0293482.s001.zip › Appendix F.doc]

Appendix F. Steps to taken to test five necessary assumptions for HLR analysis

| # | Assumption | Step | Result | Decision |
| --- | --- | --- | --- | --- |
| 1 | Normality of the data associated with the dependent variable | We computed the Mahalanobis values through the HLR in which sedentary behaviour was the dependent variable | The significance values associated with the Mahalanobis values did not meet the condition p<0.001; thus normality was not confirmed (Casson & Farmer, 2014; Asiamah et al., 2022) | We proceeded with the analysis since our sample was large (Casson & Farmer). |
| 2 | Linearity | We plotted standardized residuals against standardized predicted values of the dependent variable in the above HLR analysis. We observed the linearity of the lines of best fit. | The graph shows a straight line as recommended (Casson & Farmer, 2014) | Assumption or condition met for HLR analysis |
| 3 | Independence of errors | Durbin Watson statistics were generated for all the HLR models fitted. | Durbin-Watson statistic was approximately 2 as recommended (Casson & Farmer, 2014) | The assumption was met for HLR analysis |
| 4 | Multi-collinearity | Tolerance values were computed through the above HLR analysis | The tolerance values are >0.2 as recommended (Asiamah et al., 2022) | The assumption was met for HLR analyses |
| 5 | Homogeneity of variances | We plotted standardized residuals against standardized predicted values of the dependent variable in all HLR models | The graphs produced a satisfactory pattern as recommended elsewhere (Casson & Farmer, 2014) | The assumption was met for HLR analyses |

**Note**: HLR – hierarchical linear regression
