# Supplementary material for: Associations of frailty with partial and absolute sedentary behaviours among older adults: A STROBE-compliant analysis of modifiability by gender and age
Source: PLoS One. 2023 Oct 26;18(10):e0293482. doi: 10.1371/journal.pone.0293482 (PMC10602243; doi:10.1371/journal.pone.0293482)
Supplement: S1 Appendix — (ZIP) [file pone.0293482.s001.zip › Appendix G.doc]

Appendix G. Steps taken in the first sensitivity analyses for confounding variables

| Stage | # | Action |
| --- | --- | --- |
| 1 | 1 | Fit a simple linear regression model to assess the relationship between frailty and sedentary behaviour |
|  | 2 | Note the standardised regression weight from step 1 |
|  | 3 | Fit a multiple linear regression model in which all measured confounding variables are treated as predictors of the main independent variable, sedentary behaviour |
|  | 4 | Identify from step 3 potential confounders that have a p-value ≥0.25 |
|  | 5 | Predictors from step 4 that produced a p≥0.25 should be removed from the analysis and the others kept for the next stage of the analysis |
| 2 | 6 | Adjust for each of the remaining confounding variables in the model fitted at step 1 |
|  | 7 | Compute the per cent change between the standardised regression weight at step 1 and the new weight resulting from step 6 |
|  | 8 | All potential confounders that produce a change of 10% or more should be incorporated into the final analysis as the ultimate confounders |
