# Supplementary material for: Associations of frailty with partial and absolute sedentary behaviours among older adults: A STROBE-compliant analysis of modifiability by gender and age
Source: PLoS One. 2023 Oct 26;18(10):e0293482. doi: 10.1371/journal.pone.0293482 (PMC10602243; doi:10.1371/journal.pone.0293482)
Supplement: Attachment — Submitted filename: Response to Editor and Reviewers.doc [file pone.0293482.s003.doc]

**Response to Editor and Reviewers**

**General response** – We are deeply grateful to the editor and peer-reviewers for making time out of their busy schedules to review our manuscript. Your constructive and objective suggestions are highly appreciated, and we don’t have any objections to them. In case you feel we’ve not addressed your comments sufficiently, please we are happy to reconsider them. Please see our responses below:

Reviewer #1: This study is excellent in that it follows the STROBE checklist and confounding factors are also done in detail. It was a good learning experience for me as well as a reference for conducting my own cross-sectional study.

**Our response** – we appreciate your comment, and we are happy other expert comments from you have enabled us to enhance the manuscript.

I would like to ask you a few questions and would be happy if you could answer them.

1.[We followed a recent study [2] to measure sedentary behaviour as a construct of partial sedentary behaviour and absolute sedentary behaviour. Partial sedentary behaviour is time (in minutes) spent sitting while doing an activity such as playing a game or driving on a typical day. It was measured by asking the participants to report time spent sitting in six situations. Absolute sedentary behaviour is the time (in minutes) spent on a typical day while sitting without physically moving any part of the body. It was measured by asking the participants to report time spent while sitting in six situations (e.g., lying down, reclining, viewing TV). Sedentary behaviour was the sum of time reported on the 12 items whereas partial and absolute sedentary behaviours were the sum of their respective 6 items. Appendix A shows questions and items used to measure sedentary behaviour. Frailty was measured with the 15-item Tilburg Clinical Frailty Index (see Appendix B) with two descriptive anchors (yes – 1; no – 0) adopted in whole from a previous study [26]. As noted earlier, we used this clinical measure of frailty to be able to identify implications for clinical or geriatric practice. This scale produced a satisfactory internal consistency in the form of Cronbach’s alpha coefficient = 0.92.]

**Comment →**How much has the validity of this measure of physical activity been proven in the past? I would appreciate it if you could tell me about previous studies. Also, I thought the study would have been more reliable and valid if, in addition to the questionnaire, an objective measure (such as using an activity meter) had been taken. Why did you not use an activity meter?

**Our response** – We thank you for this. I guess you were referring to the sedentary behaviour measure. As indicated in the manuscript, this measure of sedentary behaviour was adopted from a previous study used in a similar population. Please see study/reference number 2 in the manuscript, which at least proved the predictive validity of the questionnaire. But since this questionnaire was not a Likert scale with a factor structure but captured time (in minutes) of sedentary behaviours performed, the adopted measurement was deemed appropriate though it was not validated with a traditional psychometric test. We observed that researchers don’t assess the validity and reliability of PA measures associated with time (in minutes or hours); our understanding was that reliability and validity assessments are done ideally on measures or scales with a uni- or multi-dimensional factor structure. The questionnaire used does not have a factor structure. Please we welcome any suggestions on how to handle this tricky issue, but your suggestion has unfolded an area of research that we want to consider in the near future. Regarding the activity tracker, we could not use this because of financial constraints. We have acknowledged this shortcoming in the limitations section.

2.[The participants were community-dwelling older adults aged 50 years or higher in Ghana. We targeted older adults in two peri-urban towns, each with a combination of low and high socio-economic neighbourhoods. The following inclusion criteria were used to select the participants: (1) having at least a basic education qualification (i.e., basic school living certificate), which we used as an indicator of the ability to complete the questionnaire in English; (2) being a permanent community-dwelling resident aged 50 years or higher, and (3) the ability to walk independently for at least 10 minutes [24]. We calculated the minimum sample size necessary for this study with the G*Power software and recommended statistics (i.e., effect size = 0.2, α = 0.05, power = 0.8) suited for our sample [25]. The minimum sample estimated for HLR analysis with a maximum of 10 predictors was 91. To maximise the statistical power of our tests, we selected as many eligible individuals as possible. There was no sampling frame for this study, so potential participants were selected by research assistants at community and social centres such as supermarkets and events. Research assistants interviewed potential participants at various community centres to assess their eligibility. A total of 1039 older adults who met the above criteria were selected.]

**Comment →**With regard to the eligibility of the subjects, since the study was conducted on elderly people, was any cognitive function testing done? I was concerned about the reliability of the study since it was mainly a questionnaire-based study.

**Our response** – yes and we perfectly understand your perspective. No cognitive function test was performed, but this was compensated for by our common methods bias assessment. CMB is often performed to identify any response biases due to memory problems or the respondents’ subjective/biased responses. The problem, however, was that the questionnaire used for measuring sedentary behaviour was not a Likert scale, so CMB could not be performed on it. For this reason, we ensured that only older adults with sufficient functional ability (please see inclusion criteria 3) were included in the study. This was based on the assumption that functional ability is strongly correlated with cognitive ability. However, since functional ability is not the same as cognitive ability, we have captured our inability to perform a cognitive test as a study limitation. Please the revision can be seen in the discussion section. In any case, your comment is helpful and will influence the design of our future studies.

Reviewer #2: This study uses a unique sedentary time questionnaire for the elderly and examines its correlation with frailty. Although this is an interesting study, the main concerns are listed below.

1. The introduction talks about the reliability of surveys using questionnaires for elderly people, and I think this point is important. Please add a little more detail about whether this can be compensated for by the 12-item questionnaire of this study. Furthermore, “they do not capture human "feelings or psychology, are generally expensive, and can easily be damaged", but the meaning of this part is unclear.

**Our response** – Thank you a lot, this is a helpful comment. We have indicated how our questionnaire compensates for those shortcomings. The quoted section has also been revised to make it clearer.

2. This study interprets the results based on correlation only, but has the two groups, the frailty group and the robust group, been compared? I think it would be more persuasive if this data was also shown.

**Our response** – Thanks, and this sounds like an innovative method. However, there were no such two groups (i.e., frailty group and robust group) in the study, that’s why this analysis was not done. I admit that we can create analogous groups by dichotomising the frailty variable into ‘low frailty’ and ‘high frailty’. If we were to do this, the best thing to do would be to use Three-Way ANOVA to meet the research aim, but this analysis would be less robust since it doesn’t support our sensitivity analyses. We therefore ask if we can avoid this analysis. If not, kindly give us more information to know exactly what you want us to do.
